# Type 2 Diabetes Mellitus: A Pathophysiologic Perspective

**DOI:** 10.3389/fnut.2021.707371

**Published:** 2021-08-10

**Authors:** Eric C. Westman

**Affiliations:** Department of Medicine, Duke University, Durham, NC, United States

**Keywords:** type 2 diabetes, insulin resistance, pre-diabetes, carbohydrate-restricted diets, hyperinsulinemia, hyperglycemia

## Abstract

Type 2 Diabetes Mellitus (T2DM) is characterized by chronically elevated blood glucose (hyperglycemia) and elevated blood insulin (hyperinsulinemia). When the blood glucose concentration is 100 milligrams/deciliter the bloodstream of an average adult contains about 5–10 grams of glucose. Carbohydrate-restricted diets have been used effectively to treat obesity and T2DM for over 100 years, and their effectiveness may simply be due to lowering the dietary contribution to glucose and insulin levels, which then leads to improvements in hyperglycemia and hyperinsulinemia. Treatments for T2DM that lead to improvements in glycemic control and reductions in blood insulin levels are sensible based on this pathophysiologic perspective. In this article, a pathophysiological argument for using carbohydrate restriction to treat T2DM will be made.

## Introduction

Type 2 Diabetes Mellitus (T2DM) is characterized by a persistently elevated blood glucose, or an elevation of blood glucose after a meal containing carbohydrate (1) (Table 1). Unlike Type 1 Diabetes which is characterized by a deficiency of insulin, most individuals affected by T2DM have *elevated* insulin levels (fasting and/or post glucose ingestion), unless there has been beta cell failure (2, 3). The term “insulin resistance” (IR) has been used to explain why the glucose levels remain elevated even though there is no deficiency of insulin (3, 4). Attempts to determine the etiology of IR have involved detailed examinations of molecular and intracellular pathways, with attribution of cause to fatty acid flux, but the root cause has been elusive to experts (5–7).

**Table 1:** Definition of type 2 diabetes mellitus.

**Diagnosis**	**HgbA1c**	**Fasting glucose**	**OGTT^**a**^**	**Insulin**	**Glucose in blood^**b**^**
		**mg/dL (mmol/L)**	**mg/dL (mmol/L)**	**uIU/L (pmol/L)**	**grams**
Normal	<5.7%	<100 (5.6)	<140 (7.8)	<10 (60)	<5.0
Pre-diabetes	5.7–6.4%	100–125 (5.6–6.9)	140–199 (7.8–11)	>10 (60)	5.0–6.25
Type 2 diabetes	≥6.5%	>125 (6.9)	≥200 (11.1)	>10 (60)	>6.25

a *OGGT, oral glucose tolerance test. At 2 h after drinking 75 grams of glucose*.

b *Assuming 5L of blood*.

*Source: American Diabetes Association (1)*.

## How Much Glucose Is in the Blood?

Keeping in mind that T2DM involves an elevation of blood glucose, it is important to understand how much glucose is in the blood stream to begin with, and then the factors that influence the blood glucose—both exogenous and endogenous factors. The amount of glucose in the bloodstream is carefully controlled—approximately 5–10 grams in the bloodstream at any given moment, depending upon the size of the person. To calculate this, multiply 100 milligrams/deciliter × 1 gram/1,000 milligrams × 10 deciliters/1 liter × 5 liters of blood. The “zeros cancel” and you are left with 5 grams of glucose if the individual has 5 liters of blood. Since red blood cells represent about 40% of the blood volume, and the glucose is in equilibrium, there may be an extra 40% glucose because of the red blood cell reserve (8). Adding the glucose from the serum and red blood cells totals about 5–10 grams of glucose in the entire bloodstream.

## Major Exogenous Factors That Raise the Blood Glucose

Dietary carbohydrate is the major exogenous factor that raises the blood glucose. When one considers that it is common for an American in 2021 to consume 200–300 grams of carbohydrate daily, and most of this carbohydrate is digested and absorbed as glucose, the body absorbs and delivers this glucose via the bloodstream to the cells while attempting to maintain a normal blood glucose level. Thinking of it in this way, if 200–300 grams of carbohydrates is consumed in a day, the bloodstream that holds 5–10 grams of glucose and has a concentration of 100 milligrams/deciliter, is the conduit through which 200,000–300,000 milligrams (200 grams = 200,000 milligrams) passes over the course of a day.

## Major Endogenous Factors That Raise the Blood Glucose

There are many endogenous contributors that raise the blood glucose. There are at least 3 different hormones that increase glucose levels: glucagon, epinephrine, and cortisol. These hormones increase glucose levels by increasing glycogenolysis and gluconeogenesis (9). Without any dietary carbohydrate, the normal human body can generate sufficient glucose though the mechanism of glucagon secretion, gluconeogenesis, glycogen storage and glycogenolysis (10).

## Major Exogenous Factors That Lower the Blood Glucose

A reduction in dietary carbohydrate intake can lower the blood glucose. An increase in activity or exercise usually lowers the blood glucose (11). There are many different medications, employing many mechanisms to lower the blood glucose. Medications can delay sucrose and starch absorption (alpha-glucosidase inhibitors), slow gastric emptying (GLP-1 agonists, DPP-4 inhibitors) enhance insulin secretion (sulfonylureas, meglitinides, GLP-1 agonists, DPP-4 inhibitors), reduce gluconeogenesis (biguanides), reduce insulin resistance (biguanides, thiazolidinediones), and increase urinary glucose excretion (SGLT-2 inhibitors). The use of medications will also have possible side effects.

## Major Endogenous Factors That Lower the Blood Glucose

The major endogenous mechanism to lower the blood glucose is to deliver glucose into the cells (all cells can use glucose). If the blood glucose exceeds about 180 milligrams/deciliter, then loss of glucose into the urine can occur. The blood glucose is reduced by cellular uptake using glut transporters (12). Some cells have transporters that are responsive to the presence of insulin to activate (glut4), others have transporters that do not require insulin for activation. Insulin-responsive glucose transporters in muscle cells and adipose cells lead to a reduction in glucose levels—especially after carbohydrate-containing meals (13). Exercise can increase the glucose utilization in muscle, which then increases glucose cellular uptake and reduce the blood glucose levels. During exercise, when the metabolic demands of skeletal muscle can increase more than 100-fold, and during the absorptive period (after a meal), the insulin-responsive glut4 transporters facilitate the rapid entry of glucose into muscle and adipose tissue, thereby preventing large fluctuations in blood glucose levels (13).

## Which Cells Use Glucose?

Glucose can used by all cells. A limited number of cells can *only* use glucose, and are “glucose-dependent.” It is generally accepted that the glucose-dependent cells include red blood cells, white blood cells, and cells of the renal papilla. Red blood cells have no mitochondria for beta-oxidation, so they are dependent upon glucose and glycolysis. White blood cells require glucose for the respiratory burst when fighting infections. The cells of the inner renal medulla (papilla) are under very low oxygen tension, so therefore must predominantly use glucose and glycolysis. The low oxygen tension is a result of the countercurrent mechanism of urinary concentration (14). These glucose-dependent cells have glut transporters that do not require insulin for activation—i.e., they do not need insulin to get glucose into the cells. Some cells can use glucose and ketones, but not fatty acids. The central nervous system is believed to be able to use glucose and ketones for fuel (15). Other cells can use glucose, ketones, and fatty acids for fuel. Muscle, even cardiac muscle, functions well on fatty acids and ketones (16). Muscle cells have both non-insulin-responsive and insulin-responsive (glut4) transporters (12).

## Possible Dual Role of an Insulin-Dependent Glucose-Transporter (glut4)

A common metaphor is to think of the insulin/glut transporter system as a key/lock mechanism. Common wisdom states that the purpose of insulin-responsive glut4 transporters is to facilitate glucose uptake when blood insulin levels are elevated. But, a lock serves two purposes: to let someone in *and/or* to keep someone *out*. So, one of the consequences of the insulin-responsive glut4 transporter is to keep glucose *out* of the muscle and adipose cells, too, when insulin levels are low. The cells that require glucose (“glucose-dependent”) do not need insulin to facilitate glucose entry into the cell (non-insulin-responsive transporters). In a teleological way, it would “make no sense” for cells that require glucose to have insulin-responsive glut4 transporters. Cells that require glucose have glut1, glut2, glut3, glut5 transporters—none of which are insulin-responsive (Back to the key/lock metaphor, it makes no sense to have a lock on a door that you *want* people to go through). At basal (low insulin) conditions, most glucose is used by the brain and transported by non-insulin-responsive glut1 and glut3. So, perhaps one of the functions of the insulin-responsive glucose uptake in muscle and adipose to keep glucose OUT of the these cells at basal (low insulin) conditions, so that the glucose supply can be reserved for the tissue that is glucose-dependent (blood cells, renal medulla).

## What Causes IR and T2DM?

The current commonly espoused view is that “Type 2 diabetes develops when beta-cells fail to secrete sufficient insulin to keep up with demand, usually in the context of increased insulin resistance.” (17). Somehow, the beta cells have failed in the face of insulin resistance. But what causes insulin resistance? When including the possibility that the environment may be part of the problem, is it possible that IR is an adaptive (protective) response to excess glucose availability? From the perspective that carbohydrate is not an essential nutrient and the change in foods in recent years has increased the consumption of refined sugar and flour, maybe hyperinsulinemia is the cause of IR and T2DM, as cells protect themselves from excessive glucose and insulin levels.

## Insulin Is Already Elevated in IR and T2DM

Clinical experience of most physicians using insulin to treat T2DM over time informs us that an escalation of insulin dose is commonly needed to achieve glycemic control (when carbohydrate is consumed). When more insulin is given to someone with IR, the IR seems to get worse and higher levels of insulin are needed. I have the clinical experience of treating many individuals affected by T2DM and de-prescribing insulin as it is no longer needed after consuming a diet without carbohydrate (18).

## Diets Without Carbohydrate Reverse IR and T2DM

When dietary manipulation was the only therapy for T2DM, before medications were available, a carbohydrate-restricted diet was used to treat T2DM (19–21). Clinical experience of obesity medicine physicians and a growing number of recent studies have demonstrated that carbohydrate-restricted diets reverse IR and T2DM (18, 22, 23). Other methods to achieve caloric restriction also have these effects, like calorie-restricted diets and bariatric surgery (24, 25). There may be many mechanisms by which these approaches may work: a reduction in glucose, a reduction in insulin, nutritional ketosis, a reduction in metabolic syndrome, or a reduction in inflammation (26). Though there may be many possible mechanisms, let's focus on an obvious one: a reduction in blood glucose. Let's assume for a moment that the excessive glucose and insulin leads to hyperinsulinemia and this is the cause of IR. On a carbohydrate-restricted diet, the reduction in blood glucose leads to a reduction in insulin. The reduction in insulin leads to a reduction in insulin resistance. The reduction in insulin leads to lipolysis. The resulting lowering of blood glucose, insulin and body weight reverses IR, T2DM, AND obesity. These clinical observations strongly suggest that hyperinsulinemia is a cause of IR and T2DM—not the other way around.

## What Causes Atherosclerosis?

**Figure 1:**
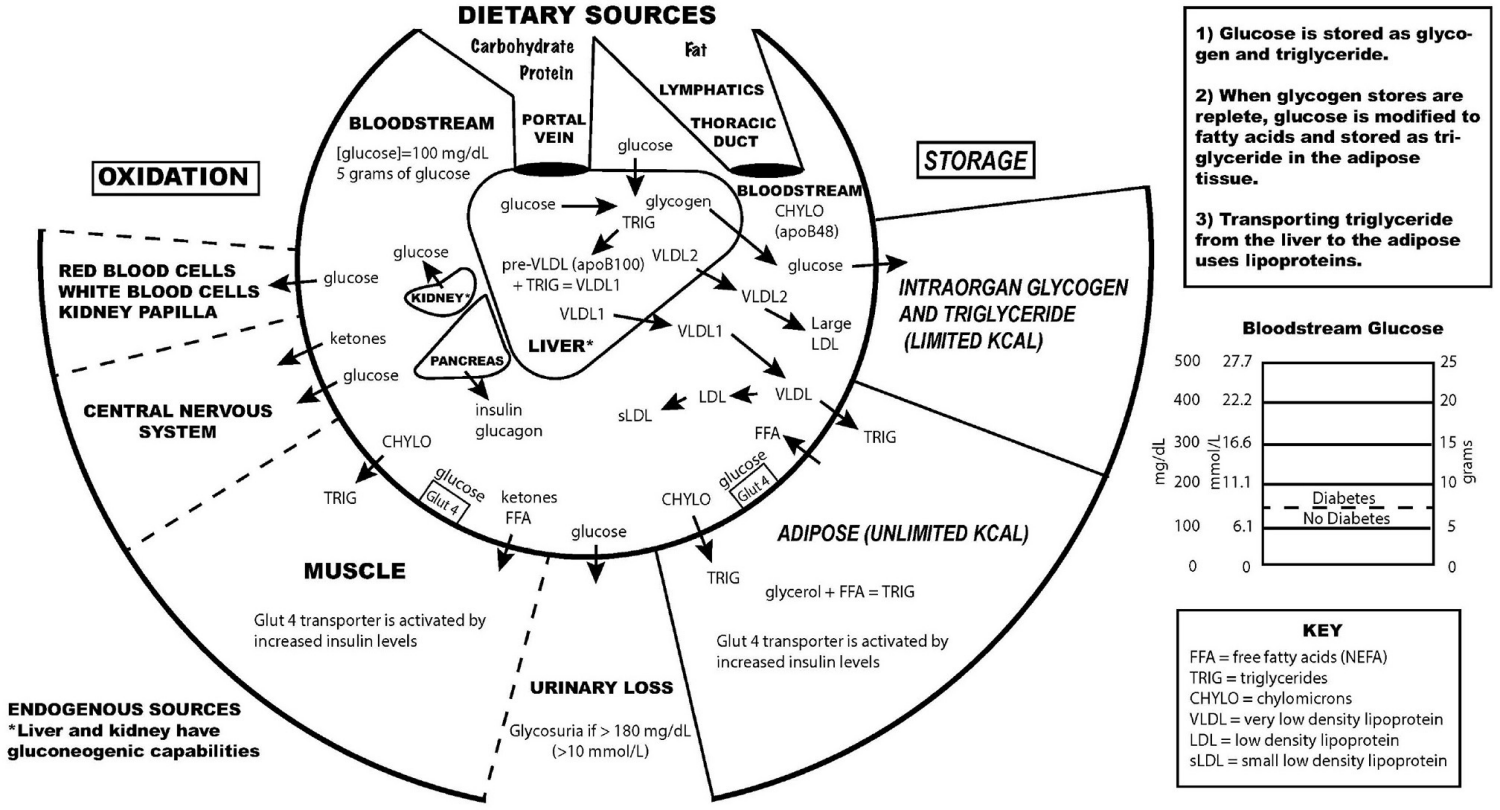
Key aspects of the interconnection between glucose and lipoprotein metabolism.

For many years, the metabolic syndrome has been described as a possible cause of atherosclerosis, but there are no RCTs directly targeting metabolic syndrome, and the current drug treatment focuses on LDL reduction, so its importance remains controversial. A recent paper compared the relative importance of many risk factors in the prediction of the first cardiac event in women, and the most powerful predictors were diabetes, metabolic syndrome, smoking, hypertension and BMI (27). The connection between dietary carbohydrate and fatty liver is well-described (28). The connection between fatty liver and atherosclerosis is well-described (29). It is very possible that the transport of excess glucose to the adipose tissue via lipoproteins creates the particles that cause the atherosclerotic damage (small LDL) (Figure 1) (30–32). This entire process of dietary carbohydrate leading to fatty liver, leading to small LDL, is reversed by a diet without carbohydrate (26, 33, 34).

## Discussion

Reducing dietary carbohydrate in the context of a low carbohydrate, ketogenic diet reduces hyperglycemia and hyperinsulinemia, IR and T2DM. In the evaluation of an individual for a glucose abnormality, measure the blood glucose and insulin levels. If the insulin level (fasting or after a glucose-containing meal) is high, do not give MORE insulin—instead, use an intervention to lower the insulin levels. Effective ways to reduce insulin resistance include lifestyle, medication, and surgical therapies (23, 35).

The search for a single cause of a complex problem is fraught with difficulty and controversy. I am not hypothesizing that excessive dietary carbohydrate is the *only* cause of IR and T2DM, but that it is a cause, and quite possibly the major cause. How did such a simple explanation get overlooked? I believe it is very possible that the reductionistic search for intracellular molecular mechanisms of IR and T2DM, the emphasis on finding pharmaceutical (rather than lifestyle) treatments, the emphasis on the treatment of high total and LDL cholesterol, and the fear of eating saturated fat may have misguided a generation of researchers and clinicians from the simple answer that dietary carbohydrate, when consumed chronically in amounts that exceeds an individual's ability to metabolize them, is the most common cause of IR, T2DM and perhaps even atherosclerosis.

While there has historically been a concern about the role of saturated fat in the diet as a cause of heart disease, most nutritional experts now cite the lack of evidence implicating dietary saturated fat as the reason for lack of concern of it in the diet (36).

The concept of comparing medications that treat IR by insulin-sensitizers or by providing insulin itself was tested in the Bari-2D study (37). Presumably in the context of consuming a standard American diet, this study found no significant difference in death rates or major cardiovascular events between strategies of insulin sensitization or insulin provision.

While lifestyle modification may be ideal to prevent or cure IR and T2DM, for many people these changes are difficult to learn and/or maintain. Future research should be directed toward improving adherence to all effective lifestyle or medication treatments. Future research is also needed to assess the effect of carbohydrate restriction on primary or secondary prevention of outcomes of cardiovascular disease.

## Data Availability Statement

The original contributions presented in the study are included in the article/supplementary material, further inquiries can be directed to the corresponding author/s.

## Author Contributions

The author confirms being the sole contributor of this work and has approved it for publication.

## Conflict of Interest

EW receives royalties from popular diet books and is founder of a company based on low-carbohydrate diet principles (Adapt Your Life, Inc.).

## Publisher's Note

All claims expressed in this article are solely those of the authors and do not necessarily represent those of their affiliated organizations, or those of the publisher, the editors and the reviewers. Any product that may be evaluated in this article, or claim that may be made by its manufacturer, is not guaranteed or endorsed by the publisher.
